# Ethical considerations of mobile phone use by patients in KwaZulu-Natal: Obstacles for mHealth?

**DOI:** 10.4102/phcfm.v6i1.607

**Published:** 2014-08-13

**Authors:** Caron L. Jack, Maurice Mars

**Affiliations:** 1 Department of TeleHealth, Nelson R Mandela School of Medicine, University of KwaZulu-Natal, South Africa

## Abstract

**Background:**

mHealth has the potential to facilitate telemedicine services, particularly in the developing world. Concern has been expressed about the confidentiality of health information that is relayed by mobile phone.

**Aim:**

We examined the habits and practices of mobile phone use by patients in KwaZulu-Natal, South Africa.

**Methods:**

We conducted a descriptive survey of two patient populations: 137 urban patients attending private practitioners and 139 patients in remote rural areas attending outpatient departments in Government-funded hospitals. The questionnaire covered several domains: demographics, mobile phone use, privacy and confidentiality and future use for health-related matters.

**Results:**

Two hundred and seventy-six patients completed the questionnaire. We found that a third of our participants shared their mobile phone with others, 24% lent their phone to others and more than half received health-related messages for other people. Mobile phone theft was common, as was number changing. Thirty-eight percent of the people were not able to afford airtime for more than a week in the past year and 22% of rural patients were unable to keep their phone charged. Mobile phone signal coverage was significantly worse in the rural areas than in urban areas.

**Conclusion:**

This study highlights the legal and ethical ramifications that these practices and findings will have on mHealth programmes in our setting. Healthcare providers and regulators will need to consider how patients use and manage their mobile phones when developing services and regulations.

## Abstrait

**Considérations d'ordre éthique sur l'usage des portables par les patients au KwaZulu-Natal: obstacles pour la santé mobile?**

**Contexte:**

La santé par téléphone portable peut faciliter les services de télémédecine, en particulier dans les pays en voie de développement. Certains craignent le manque de confidentialité des informations de santé transmises par téléphone portable.

**Objectif:**

Nous examinons les habitudes et pratiques d'utilisation des portables par les patients au KwaZulu-Natal, en Afrique du Sud.

**Méthodes:**

Nous avons mené une enquête descriptive sur deux groupes de patients: 137 patients urbains consultant des médecins privés et 139 patients dans les régions rurales éloignées en consultation externe dans les hôpitaux gouvernementaux. Le questionnaire couvrait plusieurs domaines: la démographie, l'utilisation des portables, la privauté et confidentialité, et l'utilisation future pour les questions liées à la santé.

**Résultats:**

Deux cent soixante seize patients ont rempli le questionnaire. Nous avons trouvé qu'un tiers de nos participants partageaient leur portable avec d'autres personnes, que 24% le prêtaient à d'autres et que plus de la moitié recevaient des messages liés à la santé pour d'autres personnes. Les vols de portables étaient courrants ainsi que le changement de numéro. Trente-huit pour cent des gens n'avaient pas les moyens d'acheter du temps d'appel pour plus d'une semaine au cours de l'année passée et 22% des patients ruraux ne pouvaient pas garder leur portable chargé. La réception des portables était plus mauvaise dans les régions rurales qu'en ville.

**Conclusion:**

Cette étude souligne les ramifications légales et morales que ces pratiques et résultats auront sur les programmes de santé mobile dans notre situation. Les prestataires de soins de santé et les autorités devront examiner comment les patients utilisent et gèrent leur téléphone portable dans le développement des services et règlements.

## Introduction

Telemedicine, the practice of medicine over distance using information and communication technologies, has much to offer Africa with its high burden of disease and extreme shortage of health professionals,^1^ but uptake has been disappointing.^2^ This is, in part, because of a lack of connectivity in rural areas and high telecommunication costs.^3^ Cellular or mobile phones have become a part of everyday life for many. With widespread signal coverage, they are seen as a means of facilitating telemedicine in Africa and the rest of the developing world.^4^ mHealth is the term used to describe the broad use of mobile telecommunication and multimedia technologies for the delivery of healthcare.^5^ mHealth is not another form of telemedicine, but is rather a means of data transmission using ubiquitous technology such as mobile phones and tablet computers. mHealth has, along with telemedicine, been identified as a practical solution with regard to reaching the health-related goals set out in the Millennium Development Goals (MDG) for health.^6^ These are: improving maternal health; combating HIV, malaria and other diseases; and reducing child mortality. In alignment with these MDGs, South Africa's health priorities are maternal and child health and HIV.^7^

Mobile phone penetration has grown rapidly in Africa, albeit off a very low base, to 64%.^3^ This does not mean that two-thirds of the population in Africa have mobile phones but that the number of active subscriber identity module (SIM) cards in circulation equates to two-thirds of the population. With an international average of 1.8 SIM cards per person, the proportion of people in Africa who own mobile phones was estimated to be 33% in 2012.^3^ There are also indications that mobile phone penetration in Africa is increasing at a slower rate than the rest of the developing world and may be reaching saturation.^8^ The median age in sub-Saharan Africa is 19 years and, with 43% of people living on less than USD1 per day (purchasing power parity), the cost of ownership of a mobile phone is out of reach of many people.^9^ South Africa ranks third in Africa with regard to mobile phone penetration and therefore provides a robust platform for mHealth projects.^6^

A number of studies have shown the potential benefit that mobile phone use can have for healthcare in Africa.^10, 11^ Short message service (SMS) systems have been used to remind patients of appointments^12^ and to take their medication,^11^ for health education,^13, 14^ clinical care^15^ and acquisition of data for disease surveillance,^16^ all of which have improved patient outcomes.^17, 18^

Concerns over patient confidentiality, privacy, autonomy and the security of personal and clinical data have been raised in all areas of telemedicine and medical informatics.^19^ The concepts of confidentiality and privacy are culture dependent and the importance and impact of this on mHealth solutions in Africa need further study. In the WelTel Kenyan HIV medication adherence trial, HIV-positive patients were reminded by SMS to take their medication.^20^ Patient eligibility was not dependent on owning a mobile phone but rather on having access to one or, for the illiterate, having a literate person available to take and convey the message. Confidentiality and the fear of stigma did not appear to be an issue, as a secondary analysis of the data from this trial revealed that the researchers overcame issues of confidentiality and stigma by sending a weekly check-in text message of ‘Mambo-How are you?’, requiring an active response from the participants stating that they were well or they had a problem, rather than using direct questions.^21^ In a study in Botswana on patients’ views regarding participating in a mobile phone-based dermatology service, only two of 75 people were concerned about privacy issues, but 43 people did not feel that photographs of the face were acceptable.^22^

Little has been published on confidentiality and privacy of data when using mobile phones for general clinical healthcare beyond the research arena, especially in the developing world. In thinking about the utility of mobile devices with regard to supporting patient-provider communication it is important to consider the following: (1) mobile device and network access (handset availability, capacity to keep battery charged, network availability, SIM card registration, airtime); (2) communication standards (voice or text, regulations or best practice for provider-initiated communications, availability of audit trail); and (3) sustainability (changing contact information, cost).

The aim of this study was to determine the access, availability and use of mobile devices amongst patients in KwaZulu-Natal, South Africa and thereby identify any ethical issues relating to patient–provider communication.

## Research methods and design

### Study design and setting

A descriptive, survey of two patient populations was undertaken in KwaZulu-Natal: urban patients consulting private, fee-for-service medical practitioners in Durban, a large city; and patients attending government-subsidised outpatient services in remote rural hospitals.

### Sampling strategy

The estimated sample size was 264 participants, based on the survey formula of *n* = *z* ^2^(*p*(1-*p*))/*c* ^2^, with the following parameters: 95% confidence level (*z* = 1.96), margin for error (*c* = 6%) and a worst-case percentage for picking a choice for the variables of interest (*p* = 50%). A convenience sample representing different socio-economic groups was selected.

#### Data collection

Data were collected over a period of three months. A questionnaire covering four domains, namely, patient demographics, mobile phone use, privacy and confidentiality and mobile phone use for health-related matters, was designed by the authors. The questionnaire used may be found in the Appendix. The questionnaire was piloted with several participants for validation and to check for ambiguities. Privacy and confidentiality were addressed by determining whether the respondent was the sole user of the mobile phone, whether the phone or SIM card was shared with others and if others used their SIM cards in the respondent's phone. The questionnaire also looked at mobile phone theft. Mobile phone use included issues such as financing of mobile phone calls, availability of airtime, ability to keep a mobile phone charged, sophistication of the mobile phone used, number changing and the reliability of the network signal. Health-related use addressed mobile phone use for contacting hospitals or doctors and for taking health-related messages for others. In order to maximise response rates the questionnaire was administered to the study participants by the author, with the assistance of an interpreter where necessary.

### Data analysis

The Chi Square test was used for analysis of categorical data with alpha set at 5%. Missing data were not included in the percentage and *p*-value calculations.

### Ethical considerations

The study was undertaken with the approval of the Biomedical Research Ethics Committee of the University of KwaZulu-Natal (reference number BE063/09) and verbal informed consent was obtained from the participants. All participants were over the age of 18 and no personal or identifying information was obtained.

## Results

A total of 276 people agreed to complete the questionnaire (137 urban and 139 rural patients). Thirteen of the rural responders (9.3%) did not own a mobile phone and were excluded from further analysis, leaving a total of 263 respondents, 137 urban (52%) and 126 rural (47.9%). Whilst it was envisioned that there would be two groups in the study, a third group emerged from the rural group, namely, those who work in urban areas, but reside in rural areas. The number of people in each of the three groups was as follows: urban (*n* = 137; 52.1%), rural (*n* = 83; 31.6%) and both areas (*n* = 43; 16.4%). Seventy percent of the respondents were women.

A third of all participants (*n* = 97; 36.9%) shared use of their mobile phone with others. Over half of the people (*n* = 131; 53.6%) took messages for others and 22.2% (*n* = 55) lent their phone to others. Rural respondents were significantly more likely to share SIM cards with other people and significantly more likely to be contacted by hospitals trying to contact other people (Table 1).

**TABLE 1:** Habits and practices of how people use their mobile phones.

Habit/Practice	Urban %	Rura l%	Both %	*p*
I am the only user of the phone	88/131 (67.0)	52/75 (70.7)	26/41(63.4)	-
I lend my phone to other people	26/132 (19.7)	18/75 (30.7)	11/41(26.8)	-
I share SIM cards with other people	5/132 (3.8)	10/75 (13.3)	3/41 (7.3)	0.04
Other people put their SIM card in my phone	24/125 (19.2)	18/71(25.4)	7/38 (18.4)	-
I put my SIM card in other people's phones	28/129 (21.7)	16/71 (22.5)	10/41(24.4)	-
The hospital contacts other people through my mobile phone	14/126 (11.1)	33/74 (44.6)	5/38 (13.2)	< 0.001
I take messages on my phone for other people	54/130 (41.5)	58/74 (78.4)	19/40 (47.5)	< 0.001

SIM, subscriber identity module. *p*, significant at < 0.05

Responses to questions related to connectivity, airtime and sophistication of mobile phone are shown in Table 2. Few people have mobile phone contracts and rural patients are significantly less likely to have a contract than urban patients (*n* = 3; *p* < 0001). In the past year, over a third of people (*n* = 95; 38.7%) went without airtime for more than a week, a quarter (*n* = 62; 25%) changed their mobile phone number and 23% (*n* = 58) had their mobile phone stolen. Significantly fewer rural respondents were able to keep their phones charged, with 22% reporting this as a problem (*n* = 19; *p* < 0004). Mobile phone signal coverage was significantly worse in rural areas. The rural cohort appeared to have older or simpler phones without a camera (*n* = 43; 57.3%).

**TABLE 2:** Connectivity and network issues.

Variable	Urban %	Rural %	Both %	*p* %
I buy airtime on contract	37/133 (27.8)	3/75 (4.0)	7/43 (16.3)	< 0.0001
I have been without airtime for more than a week in the last year	44/129 (34.1)	36/75 (48.0)	15/41 (36.6)	-
My number has changed in the past year	28/131 (21.4)	22/75 (29.3)	12/41 (29.3)	-
I am able to keep my battery charged	129/131 (98.5)	66/85 (78.0)	40/43 (93.0)	0.0004
I get a good phone signal at home	120/127 (94.5)	60/74 (81.1)	38/42 (90.5)	0.01
My phone has a camera	119/131 (90.8)	43/75 (57.3)	38/43 (88.7)	< 0.0001
I have had a mobile phone stolen	31/131 (23.7)	14/75 (18.7)	13/43 (30.2)	-

*p*, significant at < 0.05

Mobile phone use is shown in Table 3. Rural patients were significantly less likely to use their phones to contact their doctor (*n*= 31; *p* < 0001) or use the SMS feature (*n* = 60; *p* < 0001).

**TABLE 3:** How patients use their mobile phones.

I use my mobile phone to	Urban %	Rural %	Both %	*p*
Contact the hospital	97/130 (74.6)	49/75 (65.3)	29/41 (70.7)	N/S
Contact my doctor	108/130 (83.1)	31/75 (41.3)	33/41 (80.5)	< 0.001
SMS	125/130 (96.2)	60/75 (80.0)	39/41 (95.1)	< 0.001

*p*, significant at < 0.05

## Discussion

The study adopted a ‘bottom up’ approach to identify how patients use and manage their mobile phones and whether this would have implications on mHealth interventions within our setting. The key findings were that people in KwaZulu-Natal share mobile phones and SIM cards and take health-related messages for other people. In addition, it was found that mobile phone theft is a problem. This raises issues of possible breaches of confidentiality and privacy of patient information that could have legal and ethical implications for mHealth programmes, patients and healthcare providers if not taken into consideration.

Respect for privacy and confidentiality are seen as being fundamental human rights and are cornerstones of medical ethics, protected by law in most countries; but privacy and confidentiality are culturally-dependent concepts. Differences in the importance of privacy have been noted between Western and Japanese subjects^23^ and there have been recent changes in the understanding of privacy in China following changes in its economic policy.^24^ In most African cultures, where collective family decision-making is the norm, confidentiality is viewed in a different light.^25^ The word for confidentiality in isiZulu, the indigenous language of KwaZulu-Natal, was understood by only 30% of isiZulu-speaking people attending an outpatient clinic.^26^ In the Botswanian study on the perceptions of HIV-positive patients regarding the provision of a mobile phone-based dermatology service, few were concerned about privacy as this was guaranteed by the researchers.^22^ Kenyan HIV-positive patients received messages on shared phones and illiterate patients allowed others to receive SMS messages on their behalf which reminded them to take their medication.^20^ In South Africa, where a stigma is attached to being HIV positive, a woman was killed by her community when her HIV status became known, showing that privacy and confidentiality are of major importance.^27^ Sharing of SIM cards can allow text messages and information to be saved from a phone to the SIM card in some instances or vice versa.

Many healthcare providers (HCPs) and patients are now communicating with each other via SMS within a clinical setting.^28^ In the United Kingdom, the Medical Protection Society (MPS) has published recommendations for communicating with patients by SMS and notes that the SMS now forms part of a legal medical record. They recommend that HCPs take informed consent from patients if they are going to communicate via SMS and say that this should be noted in the patient's medical file.^29^ The recommendation does not, however, address patient-initiated SMSs. Consent is seen as a protective measure for both the HCP and the patient, providing some proof that both parties were agreeable to this form of communication and understand the risks involved.

With over a quarter of respondents in this study having changed their mobile phone number or having had their phone stolen within the past year, patient contact details would need to be verified and updated at each clinic visit. Programmes providing other SMS or mobile phone services would have to be informed of every change of number or theft. This would not, however, prevent breaches of confidentiality when messages are sent after the theft or number change but before the next clinic appointment.

Sharing of mobile phones amongst patients has also been documented in Kenya, where 41% of people were reported to share phones. Education, income, literacy, geographic location, gender and lack of electricity were cited as the primary reasons for not owning a phone.^30^ Illiteracy has been shown to have an impact on the use of SMS amongst rural South Africans, which would limit the use of SMS services to convey health information. In one rural community, the ratio of outgoing calls to SMS was 17:1.^18^ Eighty percent of rural patients in the current study use the SMS facility on their phones and 86% of rural patients want to receive medical reminders via SMS.

In rural areas, approximately 20% of people cannot keep their phones charged and do not have good signal reception. Very few have airtime contracts, with rural people being less likely to have one. Airtime is purchased on a ‘pay-as-you-go’ basis, with the most common voucher being the lowest denomination of R5.00 (± $0.50), which buys approximately four minutes of call time. Rural patients, whilst saying that they can contact the hospital using their mobile phone, indicated that they would only do so in an emergency situation requiring an ambulance as they perceive calling the hospital to be expensive. Half the rural patients were without airtime for more than a week during the past year, as were a third of the urban patients. If mHealth is to be integrated fully into the South African healthcare system, financial support or viable solutions, such as a substantial lowering of the cost of airtime for health programmes, will be needed for sustainability and cognisance will have to be taken of the fact that phones may be off the network at various times.

Over 40% of rural patients’ phones did not have a camera, suggesting that these were older, entry-level phones and the difference between the groups was significant (*p* < 0.001). It is expected that this will change as more people upgrade their current phone and their old phones filter into the rural areas. To benefit rural populations, providers of mHealth services need to offer solutions that work across a spectrum of phones, including older technologies.

In South Africa, the Health Professions Council of South Africa (HPCSA), which is the statutory body that regulates all medical practitioners and medical practice, has drafted guidelines for the practice of telemedicine. They define telemedicine as:

[*t*]he exchange of information amongst healthcare professionals at a distance for the purpose of facilitating, improving and enhancing clinical, educational and scientific healthcare and research, particularly to the under serviced areas in the Republic of South Africa.^31^

Significantly, this definition fails to mention the use of information and communication technologies, of which the mobile phone is one. The draft guidelines propose that a telemedicine consultation should only take place where there is an established patient–practitioner relationship, unless in an emergency, that all telemedicine activities require written informed consent and that written records be kept at both the send and receive site for each encounter. These requirements are problematic for mHealth services such as call centres, SMS services for general healthcare advice, education and medication reminders and telephonic consultation for a primary or second opinion. An attempt to implement a telephone-based clinical service was condemned as unethical by the HPCSA, which then also deemed telemedicine unethical because of concerns over confidentiality and privacy, amongst others.^32^ Landline telephones, however, are used routinely in clinical practice and have been since at least 1879,^33^ without the need for written informed consent and/or the requirement of written records. Regulatory consistency is thus required.

## Limitation

The use of convenience sampling may be considered to be a limitation of the study as the results may not be representative of the total population of mobile phone users in KwaZulu-Natal.

## Conclusion

It would be both prudent and ethically wise to obtain informed consent from patients in our setting prior to using mobile phones for health-related activities, as the findings of this study has identified potential breaches in maintaining the confidentiality of patient information. Consent would not, however, prevent breaches of confidentiality when phones and SIM cards are shared or when health messages are taken for other people. Obtaining consent in this instance would inform and alert the patient to these risks.

Further studies are needed in other African countries in order to determine if mobile phone use practices found in this study are common, connectivity specific, or driven by expediency or culture.
